# FAM190A Rearrangements Provide a Multitude of Individualized Tumor Signatures and Neo-antigens in Cancer

**DOI:** 10.18632/oncotarget.220

**Published:** 2011-03-02

**Authors:** Francesca Scrimieri, Eric S. Calhoun, Kalpesh Patel, Rigu Gupta, David L. Huso, Ralph H. Hruban, Scott E. Kern

**Affiliations:** ^1^ Department of Oncology, The Sidney Kimmel Comprehensive Cancer Center at Johns Hopkins, Baltimore MD 21287; ^2^ Department of Biology, Alma College, Alma, MI 48801; ^3^ Department of Molecular and Comparative Pathobiology, the Johns Hopkins Medical Institutions, Baltimore MD 21287; ^4^ Department of Pathology, the Sol Goldman Pancreatic Cancer Research Center, the Johns Hopkins Medical Institutions, Baltimore MD 21287

**Keywords:** internal rearrangements, in-frame deletion, cancer

## Abstract

We found *FAM190A* transcripts to have internal rearrangements in 40% (19/48) of unselected human cancers. Most of these tumors (84%) had in-frame structures, 94% of which involved deletion of exon 9. The *FAM190A* gene is located at 4q22.1 in a region of common fragility, FRA4F. Although normally stable in somatic cells, common fragile sites can be hotspots of rearrangement in cancer. The genomic deletion patterns observed at some sites, including FRA4F at 4q22.1, are proposed to be the result of selection for disrupted tumor-suppressor genes. Our evidence, however, indicated additional patterns for *FAM190A*. We found genomic deletions accounted for some *FAM190A* in-frame structures, and cases pre-selected for *FAM190A* genomic deletions had a yet higher prevalence of *FAM190A* rearrangements. Our evidence of widespread in-frame heterozygous and homozygous rearrangements affecting this gene in tumors of multiple types leads speculation on structural grounds that the mutant forms may retain, provide new, or possibly convey dominant-negative functions. Although a functionally uncharacterized gene, it is evolutionary conserved across vertebrates. In addition to its potential oncogenic role, the in-frame deletions predict the formation of cancer-specific *FAM190A* peptide sequences (neo-antigens) with potential diagnostic and therapeutic usefulness.

## INTRODUCTION

Common fragile sites (CFS) encompass vast chromosomal regions often containing genomically large, active genes. Although discovered by applying artificial chemical stresses to cell cultures, they can be hotspots of natural rearrangement and deletion in cancer [1]. Point mutations are rarely found in the coding region of genes within CFS [2,3]. Conversely, large intragenic homozygous deletions are frequently observed at some CFS genes, proposed to be the result of selection for disrupted tumor-suppressor genes (TSGs) such as *FHIT* and *WWOX* [4].

The analysis of the CFS DNA sequences has not clearly identified the causes of their fragility, but it was observed that they share characteristic features such as AT base richness, high degrees of DNA flexibility, and late DNA replication [5]. Despite their instability, CFSs are evolutionary stable regions as proved by their conserved features across species [6].

FRA4F is the region of common fragility at 4q22.1. Deletion at this locus was reported as a frequent event in many tumor types, suggesting 4q22.1 as the site of a not-yet-identified TSG [6,7,8,9,10]. A more recent study of esophageal cancer proposed *FAM190A* (family with sequence similarity 190, member A; *KIAA1680*; *MGC48628*), mapping to 4q22.1, as the TSG [8]. *FAM190A* is a large (1.5 Mb), functionally uncharacterized gene with no recognizable protein domain and no sequence similarity to other proteins. At least 15 different DNA sequence variants are known for *FAM190A* (http://www.ncbi.nlm.nih.gov/projects/SNP/, October 2010) none of which are associated with disease (http://www.ncbi.nlm.nih.gov/omim, October 2010).

*FAM190A* has known transcript variants (http://insdc.org/, October 2010), which share 100% identity of the 5' coding sequences to exon 6, but have different 5'and 3' UTRs and 3' exons. Variant 1 is the longest variant, containing 11 exons (NM_001145065.1); variant 2 contains 7 exons (NM_207491.2). Although the function of the protein is still unknown, the high degree of conservation across vertebrate species suggests that it has a conserved, important function.

In this study we found that the *FAM190A* transcript was very often rearranged in cancer samples. A predominance of in-frame deletions speculatively suggested a frequent activating mutation such as seen in the EGFRvIII rearrangement [11]. We suggest that the joined sequences of these in-frame deletions may form cancer-specific peptides (neo-antigens) with potential diagnostic and therapeutic relevance.

## RESULTS

### Structure of the FAM190A coding sequence

We found partially overlapping homozygous genomic deletions (HDs) of 4q22.1 in a pancreatic cancer cell line, BxPc3 (reported in ref [9]), and in two of 60 xenografted pancreatic cancers, PX19 and PX188. A fourth homozygous deletion of the same region was reported in a lung cancer line, H2126 [12], and a somatic out-of-frame deletion of two exons is reported in multiple metastases of a single pancreatic cancer [13]. In all five cases, the overlapping deleted region included the *FAM190A* gene.

In order to analyze the transcriptional pattern of *FAM190A*, overlapping primers for the human *FAM190A* transcript variant 1 were designed (see Materials and Methods and Table S1). We then performed a PCR-based screen of exons 2 to 11 on cDNAs synthesized from 72 cell lines and xenografted cancer of different types. This sample set comprised two panels: 48 unselected samples and 24 samples selected for having a known HD or small heterozygous deletions at 4q22.1 (Table S2) [7]. The gene was expressed in most samples (92%, 66/72 cases). DNA fragments of unexpected size were sequenced. In the former panel we found eight types of rearranged transcripts and internal rearrangements in nearly 40% of the cases (39.6%, 19/48 cases). Among these affected samples, 84% had in-frame structures, 94% of which involved deletion of exon 9.

**Figure 1:**
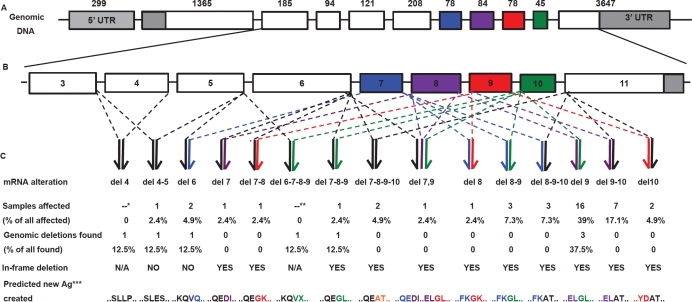
(A) The genomic structure of *FAM190A*, NM_001145065. Each box represents an exon, numbers above indicate their nucleotide lengths. Grey indicates the UTR; white and colors, the exons having nucleotide lengths not divisible and divisible by 3, respectively. (B) A magnification of exons from 3 to 11. (C) Top to bottom: structural types of the intragenic deletions, percentage of samples (combined panels) affected, percentage of homozygous deletions at the genomic level, type of rearranged transcripts, and predicted aminoacidic sequences at the transcript rearrangement joints. *cDNA not examined. **Lack of a PCR product in cDNA from exons 6 to 11. ***Predicted new antigen at the joint between normally non-contiguous exons

**Figure 2:**
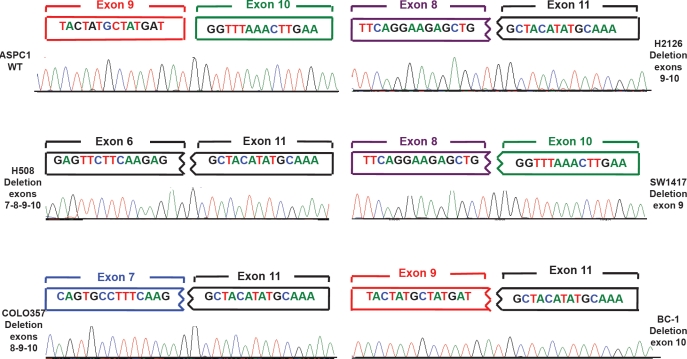
Electropherograms showing a wild-type sequence and four of the 13 different rearrangements observed For each cell line the 28bp sequence of the anomalous fusion transcript is depicted. The two boxes on the top of each diagram represent the exon at the joint. Colors are as in Figure 1.

In the latter panel, we found nine types of rearrangements and 18 aberrant cases (75%) producing exclusively in-frame structures. In 89% of the cases the deletion involved exon 9. For each case having a deleted *FAM190A* transcript, we found a co-existing spliced form of expected length (wild-type) and/or only a rearranged transcript. Among combined selected and unselected cases (Figure 1), the changes appeared homozygous in 24 samples and heterozygous in 13. Overall, we identified 13 aberrant structural transcript types between exons 2 and 11: 11 represented different in-frame deletions (Figures 1 and 2); the remaining two caused shifting of the reading frame. Two cell lines and one xenograft (H1975, SW780, and MX7) had multiple rearranged spliced forms. Sequencing of the cDNA of AsPc1 revealed no subtle (point) mutation in the coding sequence. The sequencing of the cDNA of BxPc3 revealed (beside the known deletion of exon 9 and 10) the presence of a heterozygous nonsynonymous SNP at nucleotide 1144 resulting in a missense mutation at aminoacid 382. All rearrangements could be replicated in a PCR assay using different primers. In one patient, four independent parallel xenografts (PX19-1, -2, -3, and -4) had been derived from four locations in the resected primary tumor. All four had the identical exon deletion, indicating that the deletion had pre-existed within the patient's tumor prior to expansion as xenografts.

At the protein level, the in-frame deletions were predicted to form novel peptide sequences (Table 1). These are presumptive cancer-specific “neo-antigens”.

**Table 1:** Variations in the FAM190A coding region transcripts

Deleted exons	Consequence	Predicted non-natural peptide sequence at the fusion joint*
4-5	Not in frame	SSSSKMNSL~ESFPEINKGRX †
6	Not in frame	PEFPEPSK~QVQTX
7	In frame	LKMKRVLQE~DIMKDECSM
7-8	In frame	LKMKRVLQE~GKVRHLQKA‡
7-8-9	In frame	LKMKRVLQE~GLNLKRLET
7-8-9-10	In frame	LKMKRVLQE~ATYRNRIVS
7 and 9	In frame	LKMKRVLQE~DIMKDECSMLKLQL KEKDELISQLQEEL~GLNLKRLET§,∥
8	In frame	LTEEPVPFK~GKVRHLQKA
8-9	In frame	LTEEPVPFK~GLNLKRLET††
8-9-10	In frame	LTEEPVPFK~ATYRNRIVS
9	In frame	LISQLQEEL~GLNLKRLET§
9-10	In frame	LISQLQEEL~ATYRNRIVS**
10	In frame	TQTELLCYD~ATYRNRIVS

Before the ~ sign are listed the last nine aminoacidic residues of the exon preceding the deletion. After the ~ sign are listed the first nine aminoacidic residues of the exon following the deletion. An underline indicates the aminoacidic residues that would be created due to a frame-shift. These two not-in-frame deletions would create a truncated predicted polypeptide. ^†^X, stop codon. For HLA-A*0205, the cognate ligand structure - q - g-V- -L matches del7-8^‡^ and the structure -L- - -L- -L matches the joints produced by del7 and 9^§^ and del9^§^. For HLA-A3, the ligand structure -V- - - - I-K- matches del7 and 9^∥^, the structure -L- - -L- -Y- matches del9-10^**^, and the structure - -F- -L- -K- matches del8-9^††^ [26].

The same PCR and sequencing analysis from exon 6 to exon 11 was conducted on 48 commercially available cDNAs from different normal human tissues. Reproducible expression of *FAM190A* transcripts, assessed by multiple independent replicates, was found in 37 samples, 33 of which had a wild-type *FAM190A* transcript exon structure. Four samples had an exon deletion observed once: in one case exon 9 was lost; in one exon 8; and in two, exon 7. Two samples had a cryptic intronic exon inserted, each observed once. Additional samples of the organs, however, did not confirm the observed deletion or insertion, and these were considered as unconfirmed alternative splice variants.

### Structure of FAM190A transcripts in the 5' non-coding region

5'RACE PCR was performed to determine: 1) the transcript variants expressed in our samples and 2) the structure of the *FAM190A* transcript in the 5' non-coding region. In a pancreatic cancer cell line, AsPc1, variant 1 and a novel variant 3 having an alternative first exon were observed. Based on this knowledge, RT-PCR conducted on a second pancreatic cancer cell line, BxPc3, revealed in addition to variant 1, the presence of variant 3 and a novel variant 4 having an alternative first and second exon. Variant 2 was never observed by us in the samples analyzed. In all four variants, the apparent start codon ATG is at the position 91229395 (Table 2).

**Table 2:** 5' variations in the FAM190A transcript

Variants	5'-most exon*		Additional 5' exon		5' coding exon
	Start (bp)	End (bp)	Start	End	Start ^†^
#1	91048684	91048982	-----------	----------	91229395
#2‡	91156182	91156494	-----------	----------	91229395
#3	91049688	91049813	-----------	----------	91229395
#4	91049688	91049813	91184749	91184873	91229395

^*^ The genomic positions of the exons are expressed in basepairs according to build GRCh37/hg19

^†^ The exon containing the starting codon ATG

^‡^ Variant 2 as annotated in the USCS Genome Browser (http://genome.ucsc.edu/), unconfirmed by us.

Additional unconfirmed 5′ variants are reported in the Ensembl database (http://uswest.ensembl.org/index.html).

### Structure of the genomic FAM190A

In order to rationalize the transcripts through their genomic structures, we performed PCR analysis at the intron/exon junctions of exons 4 through 10 of 45 genomic DNAs isolated from 37 cancer cell lines and eight pancreatic cancer xenografts. Among these samples, 23 had rearrangements of their transcripts. In nine, the transcript alterations were fully and/or partially explained by homozygous losses of genomic material, which encompassed only the exons deleted in the corresponding transcript. Mechanisms other than genomic deletion might underlie the aberrant transcripts in the 14 remaining cases, such as undiscovered intronic point mutations, small deletions affecting splicing signals, and heterozygous or compound heterozygous genomic deletions.

### Mouse FAM190A gene (Fam190a)

Four mouse CFSs have been defined at the molecular level. Of these, Fra6C1 corresponds to the human FRA4F [6]. The murine ortholog of the *FAM190A* gene, *Fam190a*, maps to mouse chromosome 6 and has two isoforms which differ in the 5'UTR but encode the same protein (http://www.ncbi.nlm.nih.gov/gene). The human and the mouse transcripts share 82% identity of the nucleotide coding sequence, suggesting that a deletion pattern similar to that observed in the human was possible. To test this possibility, we performed a RT-PCR-based screening analysis on two murine cancer cell lines (CT38, LLC) and one murine embryonic fibroblast line (MEF-P3) along with 36 different normal murine tissues. In 27 samples we produced an amplified fragment, which was of expected size, suggesting that no rearrangements were present. We observed widespread expression of *Fam190a* in both newborn and adult murine tissues.

## DISCUSSION

The contribution of rare and common fragile sites to genome rearrangements and diseases has long been studied. Perhaps owing to an unusual nucleotide composition and high structural flexibility, fragile sites have delayed replication in S phase, a characteristic that may lead to the formation of local replicative gaps and illegitimate chromosomal rearrangements, and result in fixed genomic deletions.

It is still not clear to what extent these play a role in cancer. Recurrent, low-frequency deletions that do not retain the reading frame can affect the coding exons of the *FHIT* and *WWOX* genes at the respective fragile sites, and intronic deletions not affecting the structure of the mature mRNA also are seen [14,15]. These patterns have lead to the controversy whether these genes may be either “driver” tumor-suppressor genes or instead reflect the uncovering of “passenger” random changes affecting fragile sites [16,17,18].

In this study we describe the finding of structural defects in the *FAM190A* transcript in 40% of human cancers and transformed cells. Evidence for widespread rearrangements affecting this region in multiple tumor types suggests that the mutant coding sequences identified might be among the most frequent mutations in human cancer. This high frequency is not readily explained by the mere coincidental location of *FAM190A* in a fragile region, for the FRA4F site spans about 10 megabasepairs, and the affected region evaluated here is less than 5% of that span. Nor does *FAM190A* have a deletion pattern in cancers similar to other altered genes evaluated at fragile sites, even if we were to restrict our attention to the exons (or groups of contiguous exons) contained in these genes in which the nucleotide count is a perfect multiple of “3”. The remaining plausible possibility is that the *FAM190A* changes of cancers is selective, wherein certain particular deletions arising from random processes has become enriched due to providing a growth advantage during neoplastic progression. This selection appears to preferentially act upon gross rearrangements, for whole-exomic and whole-genomic sequencing of human cancers (including ours) [19] has not found sub-exonic subtle mutations of this gene such as missense or nonsense mutations.

The deletions of *FAM190A* might, in theory, be recessive or dominant during tumorigenesis. Of the rearranged transcripts, 93% remained in-frame at the fusion (intragenic translocation) joint. Thirteen were apparently heterozygous, for a normal transcript co-existed with the mutant form. This suggests that the mutant protein products may retain, provide a new (or gain a) function and they are dominant. Dominant mutant genes selected during oncogenesis are classified as oncogenes.

We found that some of the in-frame rearrangements of the transcript had an obvious basis, for they corresponded to the exons spanned by intragenic homozygous deletions of the genomic DNA. In other instances, a genomic basis was implied, for the prevalence rate of *FAM190A* transcript alterations was elevated in cancers pre-selected for known heterozygous and homozygous genomic DNA deletions in the neighborhood. In the remaining tumors having no exonic genomic deletions, an undiscovered intronic mutation (similar perhaps to the genomic intronic mutations of the *CD22* gene in B-precursor leukemia proposed as causing exon 12 deletions in the transcripts) [20] or an epigenetic mechanism may be the underlying cause.

*FAM190A* has alternative transcript forms. Transcript variants can physiologically be employed to create tissue regulatory specificity or protein diversity. In particular, the presence of 5' alternative structures can derive from use of alternative promoters and/or from alternative splicing. The novel 5' variants we observed in cancer cells may represent a loss of splicing fidelity [21], may subserve a tumorigenic role, or may be shared with certain normal cells.

It will be of interest to explore the functional roles of FAM190A and how these roles may be altered by the intragenic rearrangements. The conservation of the gene among vertebrates, and especially the sharing of exon structure and exonic nucleotide lengths between human and mouse, suggests that mouse models may be fruitful to survey for the normal physiologic roles of *FAM190A*. Additionally, we noted that some of the fusion joints match the minimum consensus peptide motives presented by the MHC (Table 1). Possibly, the restricted set of fusion joints represent neo-antigens that could be clinically typed by diagnostic antibody panels, targeted by rearrangement-specific therapies, or non-invasively monitored using personalized assays for disease burden [22].

## MATERIAL AND METHODS

### Sample collection

72 human tumor specimens, 39 cell lines and 33 xenografts, were studied. 20 of the cell lines (AsPc1, BT-20, BT-474, CAPAN1, CAPAN2, CFPAC1, COLO357, DLD-1, HeLa, Hs578T, MCF7, MDA-MB-134, MDA-MB-453, MiaPaCa2, Panc-1, P215, PL45, T470, RKO, HEK 293) were randomly chosen from those available to us; the other 19 (AGS, BC-1, BxPc3, COLO205, H508, H727, HT-1376, H1581, H1975, H2126, H2228, KATO III, LNCa-Clone-FGC, LoVo, SW620, SW403, SW780, SW837,and SW1417) were selected for a having a known deletion affecting 4q22 [7]. The cell lines were obtained from European Collection of Cell Cultures (ECACC) (COLO357, P215) and American Type Culture Collection (ATCC).

33 xenografted human cancers of different types were obtained from our described tissue banks [23] under an IRB-approval protocol. Of these samples, 5 were selected for a known deletion affecting 4q22 (PX19, PX19-2R, PX19-3, PX19-4, PX188) [24] and 28 were unselected.

A panel of cDNAs from 48 different human normal tissues, was obtained (TissueScan, OriGene).

Three mouse cell lines (CT-38, LLC, MEF-P3) and a panel of 36 normal samples representing 18 different tissues taken from newborn and adult mice were studied by RT-PCR. The organs included heart, stomach, kidney, liver, lung, brain cerebellum, brain brainstem, brain cortex, pancreas, thymus, spleen, salivary gland, adrenal gland, skin, colon and small intestine.

### DNA and RNA isolation

Total RNA was extracted from cell lines and tumors (Trizol, Invitrogen). Purification was performed using columns according to manufacturer's instructions (Rneasy, Qiagen). RNA quality was assessed by gel electrophoresis of ethidium-bound total RNA. RNA was treated with DNase I (Invitrogen) and retrotranscribed (SuperScript^®^ III, Invitrogen) to form cDNA.

Genomic DNA was extracted according to manufacturer's instructions (QIAamp, Qiagen). DNA and RNA concentrations were determined using spectrometry (NanoDrop Technologies).

### Primer design, PCR, and sequence analysis

The primers were designed using Primer3 (http://frodo.wi.mit.edu/) and synthesized by Integrated DNA Technologies (IDT) (Table S3-5). Designed primers were aligned against the corresponding genome sequence using BLAT (http://genome.ucsc.edu/cgi-bin/hgBlat, assembly Feb 2009, GRCh37/hg19) to confirm specificity. Taq DNA polymerase was used for the PCR reactions.

PCR conditions were as follows: 94 °C for 4 min, 72°C for 10 s, and then 40 cycles of 94 °C for 30 s, 55°C for 30 s, 72°C for 30 s. PCR products were separated on 1% agarose gel in lithium boric acid buffer (LB®, Faster Better Media LLC) [25] to determine presence and size, processed (QIAquick PCR Purification Kit, Qiagen) and analyzed by automated sequencing.

### RACE PCR

5'RACE PCR was performed (FirstChoice RLM-RACE, Applied Biosystem) according to manufacturer's instructions.

## SUPPLEMENTAL TABLES










