# Supplementary material for: FAM190A Rearrangements Provide a Multitude of Individualized Tumor Signatures and Neo-antigens in Cancer
Source: Oncotarget. 2011 Mar 2;2(1-2):69–75. doi: 10.18632/oncotarget.220 (PMC3167148; doi:10.18632/oncotarget.220)
Supplement: Supplementary file 1 [file oncotarget-02-069-s001.docx]

**Table S1: cDNAs primer sequences used in the PCR-based analysis**

| **Primer name** | **Sequence** | **Chromosomal position (Start bp)*** | **Target** |
| --- | --- | --- | --- |
| FAM190A-01F | ATCCGCCTCTCCTCTCTCTC | + 91048709 | 5’UTR |
| FAM190A-01R | CCCTTCACTTTTGCCAGAAC | - 91229893 | Exon 2 |
| FAM190A-02F | TGAAGGGGATGATTCTGGTT | +91229906 | Exon 2 |
| FAM190A-02R | CCAAGTTTCCTTGGGGAGTT | - 91230600 | Exon 2 |
| FAM190A-03F | CCCCAAGGAAACTTGGATTT | + 91230604 | Exon 2 |
| FAM190A-03R | GAAAGGCACTGGCTCTTCAG | - 91645120 | Exon 7 |
| FAM190A-04F | GATGAATGCTCGATGCTCAA | + 91736925 | Exon 8 |
| FAM190A-04R | TCCTGCTGGCCTAGAGTTTG | -92520182 | Exon 11 |
| FAM190A-05F | AGCAATCTGTTCACTGTTAATGG | + 92520556 | 3’UTR |
| FAM190A-05R | TTTGCCATCAAATGGGTTTT | - 92521322 | 3’UTR |
| FAM190A-06F | TTTGACAAATAAGGCCCAAA | + 92521350 | 3’UTR |
| FAM190A-06R | TAAATATTGCCCAGCCCAAA | - 92522087 | 3’UTR |
| FAM190A-07F | CAGGCCTGGAGAAGGTGTAA | + 92522584 | 3’UTR |
| FAM190A-07R | GGGGCAACTATGCCTACAAA | - 92523331 | 3’UTR |
| FAM190A-08F | TTTGCGTGTTTGTAATTCTGC | + 92521964 | 3’UTR |
| FAM190A-08R | CACTGAAAGAATGCCCACAT | - 92522693 | 3’UTR |
| FAM190A-09F | CCTTTACAGGCAGGTTTGGA | + 92520078 | Exon 11 |
| FAM190A-09R | CAAGCAAGCCACATTCTTCA | - 92520705 | 3’UTR |
| FAM190A-10F | TGAAGAATGTGGCTTGCTTG | + 92520705 | 3’UTR |
| FAM190A-11R | TTGAGTGCTCTTTTTCCCTTTC | -91229841 | Exon 2 |
| FAM190A-12F | AGGGGAAGACATTCTGTTGG | + 91229769 | Exon 2 |
| FAM190A-12R | GAGTTTGTCCCCAGCATTGT | - 91230585 | Exon 2 |
| FAM190A-13F | GAAGGCACTGCAGGGAGTAG | + 91234082 | Exon 3 |
| FAM190A-14F | GCAGACATGAGTCCAGCAAG | + 91645068 | Exon 7 |
| FAM190A-15F | GTCAGCCGTAAGCTGAAACC | + 91049722 | 5’UTR** |
| FAM190A-15R | GGGAGACCAGGGTAGATCGT | - 91229456 | Exon 2 |
| FAM190A-16F | ACAATGCTGGGGACAAACTC | + 91230585 | Exon 2 |
| FAM190A-16R | TACTCCCTGCAGTGCCTTCT | - 91234081 | Exon 3 |
| FAM190A-17F | CCCAACTTCAGGAAGAGCTG | + 91736977 | Exon 8 |
| FAM190A-17R | TTTTGTAGGGGCTTGTCTGG | - 92519824 | Exon 11 |
| FAM190A-18F | AATCTGTCAGCGTGCACAAG | + 92521643 | 3’UTR |
| FAM190A-18R | GCACATGTGTGTTCCTTTGG | - 92521911 | 3’UTR |
| FAM190A-19F | CATCTAAAGTTCCCACCATAC | + 92522033 | 3’UTR |
| FAM190A-19R | ACAGACACCAACATTTAGGAC | - 92522165 | 3’UTR |
| FAM190A-20F | GAATGCCCAACAGTCCATCT | + 91549238 | Exon 6 |
| FAM190A-21R | CGTCTGAAGTGAGGGTTTCA | - 92519935 | Exon 11 |
| FAM190A-22F | GAGTCCAGCAAGCAGTACCA | + 91645076 | Exon 7 |
| FAM190A-23R | AGAACGTGGAGAGCCCTTCT | -92520013 | Exon 11 |
| FAM190A-24F | GGCCTCTACAAGGTGTGGAA | + 91549265 | Exon 6 |
| FAM190A-24R | CCGTAGCAACTTGGTCCTTT | - 92520043 | Exon 11 |
| FAM190A-25R | GTGGCCCGTAAGTTCTGTGT | - 92519957 | Exon 11 |

* Chromosomal position refers to GRCh37/hg19

**Refers to isoforms 3 and 4

When PCR product was sequenced, same primers used to amplify the amplicon were used.
