# Supplementary material for: FAM190A Rearrangements Provide a Multitude of Individualized Tumor Signatures and Neo-antigens in Cancer
Source: Oncotarget. 2011 Mar 2;2(1-2):69–75. doi: 10.18632/oncotarget.220 (PMC3167148; doi:10.18632/oncotarget.220)
Supplement: Supplementary file 2 [file oncotarget-02-069-s002.docx]

**Table S2: Human cell lines used for the analysis of FAM190A transcripts.**

| **Cancer cell line** | **Tissue of origene** | **Disease** | **Type of transcript** | **Selected/Unselected** |
| --- | --- | --- | --- | --- |
| AGS | Stomach | Gastric Adenocarcinoma | Deletion exon 10 | Selected |
| AsPc1 | Pancreas | Adenocarcinoma | WT* | Unselected |
| BC-1 | Blood, B lymphocyte | Lymphoma | Deletion exon 10 | Selected |
| BT-20 | Breast | Carcinoma | Deletion exon 6 | Unselected |
| BT-474 | Breast | Ductal carcinoma | WT | Unselected |
| BxPc3 | Pancreas | Adenocarcinoma | Deletion exons 9-10 | Selected |
| CAPAN1 | Pancreas | Adenocarcinoma | WT | Unselected |
| CAPAN2 | Pancreas | Adenocarcinoma | WT | Unselected |
| CFPAC1 | Pancreas | Ductal adenocarcinoma/ cystic fibrosis | WT | Unselected |
| COLO205 | Colon | Colorectal adenocarcinoma | Deletion exons 8-9-10 | Selected |
| COLO357 |  |  | Deletion exons 8-9-10 | Unselected |
| DLD-1 | Colon | Colorectal adenocarcinoma | WT | Unselected |
| H508 | Cecum | Colorectal adenocarcinoma | Deletion exons 7-8-9-10 | Selected |
| H727 | Lung | Carcinoid | WT | Selected |
| H1581 | Lung | Non-small cell lung cancer | WT | Selected |
| H1975 | Lung | Adenocarcinoma/ Non-small cell lung cancer | Deletion exon 8, exons 8-9, exons 8-9-10 | Selected |
| H2126 | Lung | Adenocarcinoma/ Non-small cell lung cancer | Deletion exons 9-10 | Selected |
| H2228 | Lung | Adenocarcinoma/ Non-small cell lung cancer | Deletion exons 8-9 | Selected |
| HEK 293 | Embryonic kidney |  | Deletion exon 7-8 | Unselected |
| HeLa | Cervix | Adenocarcinoma | WT | Unselected |
| Hs 578T | Breast | Carcinoma | Undetected | Unselected |
| HT-1376 | Urinary bladder | Carcinoma | Undetected | Selected |
| KATO III | Stomach | Gastric carcinoma | WT, deletion exons 9-10 | Selected |
| LNCA-CLONE-FGC | Prostate | Adenocarcinoma | Undetected | Selected |
| LoVo | Colon | Colorectal adenocarcinoma | Undetected | Selected |
| MCF7 | Breast | Adenocarcinoma | WT | Unselected |
| MDA-MB-134-VI | Breast | Ductal carcinoma | WT | Unselected |
| MDA-MB-453 | Breast | Metastatic carcinoma | WT | Unselected |
| MiaPaCa2 | Pancreas | Carcinoma | Undetected | Unselected |
| Panc-1 | Pancreas | Epithelioid carcinoma | Deletion exons 4-5 | Unselected |
| P215 | Pancreas | Ductal carcinoma | WT | Unselected |
| PL45 | Pancreas | Ductal carcinoma | WT | Unselected |
| RKO | Colon | Carcinoma | Deletion exon 6 | Unselected |
| SW1417 | Colon | Colorectal adenocarcinoma | Deletion exon 9 | Selected |
| SW403 | Colon | Colorectal adenocarcinoma | Deletion exons 7-8-9 | Selected |
| SW620 | Colon | Colorectal adenocarcinoma | Undetected | Selected |
| SW780 | Urinary bladder | Transitional cell carcinoma | WT, deletion exons 7,9, deletion exon 9 | Selected |
| SW837 | Rectum | Adenocarcinoma | Deletion exons 7-8-9-10 | Selected |
| T47D | Breast | Ductal carcinoma | WT, deletion exon 9 | Unselected |

*WT: wild-type
