# Supplementary material for: FAM190A Rearrangements Provide a Multitude of Individualized Tumor Signatures and Neo-antigens in Cancer
Source: Oncotarget. 2011 Mar 2;2(1-2):69–75. doi: 10.18632/oncotarget.220 (PMC3167148; doi:10.18632/oncotarget.220)
Supplement: Supplementary file 3 [file oncotarget-02-069-s003.docx]

**Table S3: gDNAs primer sequences used in the PCR-based analysis**

| **Primer Name** | **Sequence** | **Chromosomal Position**  **(Start bp)*** | **Target** |
| --- | --- | --- | --- |
| gFAM190A-01F | gggcatttacccagacagaa | +91228982 | Intron 1 |
| gFAM190A-01R | TTTGCAGCctgagaaaggtt | -91229383 | Exon 2/ Intron 2 |
| gFAM190A-02F | ACAATGCTGGGGACAAACTC | +91230585 | Exon 2 |
| gFAM190A-02R | aaaatcaagcaggggaaaca | -91230907 | Intron 2 |
| gFAM190A-03F | ATCCTGCCAAAGgtatgctg | +91230748 | Exon 2/Intron 2 |
| gFAM190A-03R | tttcttctgaaggtgctccaa | -91231036 | Intron 2 |
| gFAM190A-04F | gtgagcaacgaagcaacaaa | +91233797 | Intron 2 |
| gFAM190A-04R | TACTCCCTGCAGTGCCTTCT | -91234081 | Exon 3 |
| gFAM190A-05F | ATCCTCGTCAGAAGGCACTG | +91234072 | Exon 3 |
| gFAM190A-05R | tggaaacaaattttcccttca | -91234306 | Intron 3 |
| gFAM190A-06F | AAGCAAAGAGCAGGTTCTTCA | +91234157 | Exon 3 |
| gFAM190A-06R | tccatggaagcaaggaagac | -91234408 | Inton 3 |
| gFAM190A-07F | catttttgcttcacggcttc | +91321099 | Intron 3 |
| gFAM190A-07R | gaaagctactgaccagggtagg | - 91321316 | Intron 4 |
| gFAM190A-08F | TTGGGATCTTGTGAACTGGA | +91321202 | Exon 4 |
| gFAM190A-08R | cgcacccaaattatgtctcc | - 91321428 | Intron 4 |
| gFAM190A-09F | aatgacttgagggagcctga | +91321825 | Intron 4 |
| gFAM190A-09R | gcagtaggtcccaacgttct | - 91322063 | Intron 4 |
| gFAM190A-10F | tgttttcctgcccattcttc | +91320536 | Intron 4 |
| gFAM190A-10R | aaaggaatttgctcactgcaa | - 91320784 | Intron 4 |
| gFAM190A-11F | gaaaaattgcccaaggtcat | +91389191 | Intron 4 |
| gFAM190A-11R | AACTACTGCGGCACATGAGA | -91389415 | Exon 5 |
| gFAM190A-12F | aaggggaaaaaggagatgtga | +91389311 | Intron 4 |
| gFAM190A-12R | ctcacTGTTTGGAAGGCTCA | - 91389491 | Intron 4/Exon 5 |
| gFAM190A-13F | aggaaggagaatcccttgga | +91388736 | Intron 4 |
| gFAM190A-13R | tttgcaacatgaccacacct | -91389055 | Intron 4 |
| gFAM190A-14F | ggtgtggtcatgttgcaaag | +91389056 | Intron 4 |
| gFAM190A-14R | acTGTTTGGAAGGCTCAGGA | -91389488 | Exon 5/Intron 5 |
| gFAM190A-15F | CGTTGATCAAGAAGCCAGGT | +91549203 | Exon 6 |
| gFAM190A-16F | tgctctaagcatgctgcact | +91549124 | Intron5 |
| gFAM190A-16R | gagcagggtctaaccacgtc | - 91549441 | Intron 6 |
| gFAM190A-17F | GAATGCCCAACAGTCCATCT | +91549238 | Exon 6 |
| gFAM190A-18F | GTGCAGACATGAGTCCAGCA | +91645066 | Exon 7 |
| gFAM190A-18R | atttgaaatcagacccttgaaga | - 91645243 | Intron 7 |
| gFAM190A-19F | tgcacttaaaatatacatggagagtga | +91644829 | Intron 6 |
| gFAM190A-19R | GAAAGGCACTGGCTCTTCAG | -91645120 | Exon 7 |
| gFAM190A-20F | ctcccactgtgtgaagctga | +91736827 | Intron 7 |
| gFAM190A-20R | ggattgaaccactggaagga | - 91737098 | Intron 8 |
| gFAM190A-21F | tcaagagcaaagtaaaagggcta | +91736856 | Intron 7 |
| gFAM190A-21R | aaccgaaatgtttaatcctggtt | - 91737235 | Intron 8 |
| gFAM190A-22F | GATGAATGCTCGATGCTCAA | +91736925 | Exon 8 |
| gFAM190A-22R | acctgaaccacacacactgc | -91737499 | Intron 8 |
| gFAM190A-23F | CCCAACTTCAGGAAGAGCTG | +91736977 | Exon 8 |
| gFAM190A-23R | aatgtggcctaagccttcaa | -91737274 | Intron 8 |
| gFAM190A-24F | aaggtgggacagttgtgacc | +91844327 | Intron 8 |
| gFAM190A-24R | ccaggaggtatttggggatt | - 91844738 | Intron 9 |
| gFAM190A-25F | cattttgcagGGAAAAGTCC | +91844511 | Intron 8/Exon 9 |
| gFAM190A-25R | gtgcaccgctaaatcctgtt | -91844696 | Intron 9 |
| gFAM190A-26F | TACTCAGGGCCATCGCTTAG | +92015880 | Intron 10 |
| gFAM190A-26R | TAATTGGAGCAACGGGAGTC | - 92016170 | Intron 10 |
| gFAM190A-27F | gtactgcaagggcttctgct | +92006680 | Intron 9 |
| gFAM190A-28F | gcgctgagatctgctggtag | +92006727 | Intron 9 |
| gFAM190A-28R | tgctttggcagaatgaacag | - 92007288 | Intron10 |
| gFAM190A-29F | caggaaatgcattgggacta | +92006894 | Intron 9 |
| gFAM190A-29R | ttttcccacccctcttcttt | -92007158 | Intron10 |

*Chromosomal position refers to GRCh37/hg19

Small nd capital letters refer to intronic and exonic sequence, respectively.
