# Supplementary material for: FAM190A Rearrangements Provide a Multitude of Individualized Tumor Signatures and Neo-antigens in Cancer
Source: Oncotarget. 2011 Mar 2;2(1-2):69–75. doi: 10.18632/oncotarget.220 (PMC3167148; doi:10.18632/oncotarget.220)
Supplement: Supplementary file 4 [file oncotarget-02-069-s004.docx]

**Table S4: cDNA primer sequences used in the 5’RACE analysis**

| **Primer Name** | **Sequence** | **Chromosomal Position (Start) bp** | **Target** |
| --- | --- | --- | --- |
| RACEFAM190A-01R | GGCTCTTGCTTAGGCTCACTCCCCTTC | - 91229654 | Exon 2 |
| RACEFAM190A-02R | TATTGGCAACCGGGAGACCAGGGTAG | -91229461 | Exon 2 |
| FAM190A-01R | CCCTTCACTTTTGCCAGAAC | - 91229893 | Exon 2 |
