# Supplementary material for: FAM190A Rearrangements Provide a Multitude of Individualized Tumor Signatures and Neo-antigens in Cancer
Source: Oncotarget. 2011 Mar 2;2(1-2):69–75. doi: 10.18632/oncotarget.220 (PMC3167148; doi:10.18632/oncotarget.220)
Supplement: Supplementary file 5 [file oncotarget-02-069-s005.docx]

**Table S5: cDNA mouse primer sequences used in the RCR-based analysis**

| **Primer Name** | **Sequence** | **Chromosomal position**  **(Start) bp*** | **Target** |
| --- | --- | --- | --- |
| mFam190a1F | CCAGTGGATCCAGGAAAAGA | +61373007 | Exon 5 |
| mFam190a1R | TGGTTCGATTTCGGTGTGTA | - 62329781 | Exon 11 |
| mFam190a2F | CTTGCCAGTAGCCTCAGTCC | 61263650 | Exon 3 |
| mFam190a2R | CAGAAGCAGCGTCTTCACTG | - 61520828 | Exon 6 |
| mFam190a3F | CGCTCAGAATGACTGACTGC | +61130464 | 5’ UTR |
| mFam190a3R | CCTGGTTTTGACGTGTTCCT | - 61261165 | Exon 2 |
| mFam190a4F | GCAGGAGGAGTGCTGAAGAC | +61130525 | 5’ UTR |
| mFam190a4R | TGTCTTCCCCTGGTTTTGAC | - 61261173 | Exon 2 |
| mFam190a5F | ACCACCTCACTTCCCATCAG | +61130525 | Exon 7 |
| mFam190a5R | TGGGTCCTTTGGTTTTTCTG | - 62330086 | Exon 11 |
| mFam190a6R | GATGGGGAAGGTCTCCCTAA | -61261515 | Exon 2 |
| mFam190a7F | TAGGGAGACCTTCCCCATCT | +61261516 | Exon 2 |
| mFam190a7R | CCAGTTCACAGGATCCCAGA | 61323969 | Exon 4 |
| mFam190a8F | AACGCTTTAAAGGGGTCCAC | 61262059 | Exon 1 |
| mFam190a8R | TCTTGAAGCAAACGCCTTCT | 61760644 | Exon 8 |
| mFam190a9F | TGCCATTCAGACTGATGCTC | 61520795 | Exon 5 |
| mFam190a9R | GGGCATGACCACTCTTAGGT | 62330311 | 3’UTR |
| mFam190a10F | AGAGTGATGTGAGCCCTTCC | 61588431 | Exon6/7 |
| mFam190a10R | TCAGTCAACATACTCAAGCTGTCA | 62330395 | 3’UTR |
| mFam190a11F | AGTGAAGACGCTGCTTCTGA | 61520829 | Exon 6 |
| mFam190a11R | AAAAGCCCATAAGTCTTTGCT | 62330358 | 3’UTR |
| mActb1F | CTGTATTCCCCTCCATCGTG | -143667261** |  |
| mActb1R | AAGGAAGGCTGGAAAAGAGC | + 143666028** |  |

*Chromosomal position according to NCBI37/mm9

** Actb is on chromosome 5
